# Flow Cytometry and Its Applications to Molecular Biology and Diagnosis 2.0

**DOI:** 10.3390/ijms242216215

**Published:** 2023-11-11

**Authors:** Stefano Papa, Claudio Ortolani, Paula Fernández, José-Enrique O’Connor

**Affiliations:** 1 Department of Biomolecular Sciences, University of Urbino Carlo Bo, 61029 Urbino, Italy; stefano.papa@uniurb.it (S.P.);; 2 Institute for Laboratory Medicine, Kantonsspital Aarau, 5001 Aarau, Switzerland; paula.fernandez@ksa.ch; 3 Laboratory of Cytomics, Joint Research Unit CIPF-UVEG, The University of Valencia and Principe Felipe Research Center, 46010 Valencia, Spain

Flow cytometry is a single-cell based technology aimed to quantify the scattering of light and the emission of multiple fluorescence signals by individual cells, biological vesicles, or synthetic microscopical particles when examined one by one at high speed using lasers or other suitable illumination sources [1,2]. In conventional hydrodynamical flow cytometers, cells or particles are suspended in an appropriate liquid medium and focused using the illumination system, thus enabling a sophisticated optical system to simultaneously quantify different structural and functional properties of each cell or particle at thousands of events per second. The functional characterization of cellular parameters mostly derives from fluorescence-based analysis, but other forms of interaction with light provide relevant information on cell structure and morphology. The computer-integrated data matrix provides a comprehensive description of the biological features of the sample. The increasing availability of fluorescent reagents and the recent development of algorithms for multispectral fluorescence unmixing allows for the simultaneous determination of up to 30–50 parameters per cell [3]. The capability to identify cell subpopulations, including very rare cells, makes flow cytometry an essential technology for clinical diagnosis and prognosis, especially in the fields of immunology [4] and onco-hematology [5]. Moreover, functional and real-time analysis makes flow cytometry a relevant tool in the fields of cellular and molecular biology, biotechnology, toxicology and drug discovery, and environmental studies [6].

This new Special Issue entitled “Flow Cytometry and Its Applications to Molecular Biology and Diagnosis 2.0” of the International Journal of Molecular Sciences includes a total of five contributions: three original articles and two reviews, providing new information about advanced applications of flow cytometry in the fields of molecular and cell biology for basic and translational research.

Rossi et al. [7] extensively reviewed the most recent advances in the application of flow cytometry to the analysis of gene expression in studies of immunometabolism. This is a growing field aimed to define how metabolic pathways affect immune functions. Multiparametric flow cytometry permits the analysis RNA expression at the single-cell level, combined with cytometric determinations of surface and intracellular markers and of metabolic processes. In their review [7], Rossi et al. discussed the technical bases of RNA flow cytometry and how it can be combined with conventional flow cytometry to provide a complete description of the immunometabolic profile of T cells at the single-cell level. This was exemplified by a study on regulatory T cells (Tregs), in which RNA flow cytometry was shown to be compatible with the analysis of intranuclear proteins and of mitochondrial mass.

Drug development has evolved rapidly, and in the last years, cell and gene therapies are becoming efficient alternatives to small chemical and biomolecule pharmacology. Several cell and gene therapies have reached regulatory approval, specially in the field of tumor immunotherapy, and such a breakthrough demands suitable bioanalytical techniques for fully characterizing the pharmacokinetics of these therapies. However, these different techniques may not always lead to concordant results, and this fact requires a deep understanding of the technical grounds of each technique, which data are generated, and how these data can be compared and interpreted. In their review, Hays et al. [8] critically compared two industry-relevant assay platforms for measuring cellular kinetics in cell therapies that express unique transgenes, namely quantitative PCR (qPCR) and multiparametric flow cytometry. Through evaluation of the pros and cons of each platform and proposing regulatory assay guidelines, Hays et al. showed that qPCR is superior in its sensitivity, while multiparametric flow cytometry provides a direct measure and characterization of the cell therapy product. Moreover, flow cytometry can resolve viable cell therapy phenotypic subsets and reveals the physiological state of cell therapy.

Among the new directions for cancer therapy, nanomaterials appear as a promising tool for drug delivery into specific cells or subcellular compartments. In this regard, fluorescent silica nanoparticles (SiNPs) appear to be a promising imaging platform, as they show intramitochondrial colocalization in biological cell models and can be used as efficient drug delivery systems when conjugated to a therapeutic molecule. Sola et al. [9] performed an experimental study aimed to characterize the intracellular targeting of SiNPs in myeloid cell lines and the cytotoxicity and cellular effects of doxorubicin-conjugated SiNPs (DOX-NPs) on the breast cancer line MCF-7. Their results indicated that DOX-NPs are endocytosed and co-localize with lysosomes, inducing a cell cycle arrest and promoting apoptosis. Moreover, the DOX-NPs induced a gradual decrease in CD44 expression on the cell surface and a minor release of CD44^+^ extracellular vesicles (EVs). These results support that the conjugation of antitumor drugs to SiNPs is an efficient way for drug delivery that potentiates drug cytotoxicity, while reducing side effects.

The paper by Montanari et al. [10] explored the biotherapeutic potential of EV secretion as a tool for delivering bacteria-derived antitumor compounds, such as the cytolethal distending toxin (CDT) produced by *Campylobacter jejuni* and other Gram-negative bacteria. In the first part of their in vitro experimental design, Montanari et al. showed that CDT-containing bacterial lysates induce lethal and sublethal alterations in intestinal (CaCo-2) and myeloid (U937) cell lines, including cell cycle arrest, mitochondrial dysfunction, and oxidative stress. These intracellular changes were accompanied by relevant changes in lysosomal exocytosis, secretory autophagy, and EV release. Based on these effect on the secretion processes, Montanari et al. investigated the presence of active CDT in EVs secreted by CDT-treated CaCo-2 cells and found that CDT-like effects were transferred from infected CaCo-2 cells to uninfected heterologous (U937) and homologous (CaCo-2) cells. Their results suggest that EVs from CDT-treated CaCo-2 cells are reliable CDT carriers and could potentially be used in the treatment of colorectal cancer.

Analysis of reactive oxygen species (ROS) and oxidative stress is a very relevant application of flow cytometry. However, to ascertain the specific role of ROS in oxidative stress studies via cytomic methodologies, it is essential to detect and characterize these species accurately. While there is a large availability of fluorescent probes for ROS analysis via flow cytometry, these reagents exhibit important limitations in their specificity and sensitivity that may affect the accuracy of the analysis. Jávega et al. [11] presented a flow cytometric study aimed to validate a new experimental model based on *Escherichia coli* B strains deficient in key genes of the antioxidant defense, namely *oxyR*, *sodA,* and *sod B*. Jávega et al. applied this model for systematically assessing issues of ROS specificity of several relevant fluorescent probes and the involvement of different ROS in the oxidative stress induced on these strains by exogenous prooxidants. Their results suggest that the antioxidant-deficient *Escherichia coli* B strains can be used as ROS biosensors in flow cytometric studies of oxidative stress and confirm the specificity issues of ROS-sensitive fluorescence that have already been detected in eukaryotic models. In addition, Jávega et al. provided recommendations for the proper design of flow cytometric studies involving ROS detection.
